# Genome-Based Classification of Strain 16-SW-7, a Marine Bacterium Capable of Converting B Red Blood Cells, as *Pseudoalteromonas distincta* and Proposal to Reclassify *Pseudoalteromonas paragorgicola* as a Later Heterotypic Synonym of *Pseudoalteromonas distincta*

**DOI:** 10.3389/fmicb.2021.809431

**Published:** 2022-02-08

**Authors:** Olga I. Nedashkovkaya, Song-Gun Kim, Larissa A. Balabanova, Natalia V. Zhukova, Oksana M. Son, Liudmila A. Tekutyeva, Valery V. Mikhailov

**Affiliations:** ^1^G.B. Elyakov Pacific Institute of Bioorganic Chemistry, Far Eastern Branch of the Russian Academy of Sciences, Vladivostok, Russia; ^2^Korean Collection for Type Cultures, Biological Resource Center, Korea Research Institute of Bioscience and Biotechnology, Daejeon, South Korea; ^3^A.V. Zhirmunsky National Scientific Center of Marine Biology, Far Eastern Branch of the Russian Academy of Sciences, Vladivostok, Russia; ^4^Department of Bioeconomy and Food Security, School of Economics and Management, Far Eastern Federal University, Vladivostok, Russia

**Keywords:** marine bacteria, taxonogenomics, phenotype, emended description of *Pseudoalteromonas distincta*, *Pseudoalteromonas paragorgicola*

## Abstract

A strictly aerobic, Gram-stain-negative, rod-shaped, and motile bacterium, designated strain 16-SW-7, isolated from a seawater sample, was investigated in detail due to its ability to produce a unique α-galactosidase converting B red blood cells into the universal type blood cells. The phylogenetic analysis based on 16S rRNA gene sequences revealed that the strain 16-SW-7 is a member of the *Gammaproteobacteria* genus *Pseudoalteromonas*. The closest relatives of the environmental isolate were *Pseudoalteromonas distincta* KMM 638^T^ and *Pseudoalteromonas paragorgicola* KMM 3548^T^, with the plural paralogous 16S rRNA genes of 99.87–100% similarity. The strain 16-SW-7 grew with 1–10% NaCl and at 4–34°C, and hydrolyzed casein, gelatin, tyrosine, and DNA. The genomic DNA G+C content was 39.3 mol%. The prevalent fatty acids were C_16:1_ ω7*c*, C_16:0_, C_17:1_ ω8*c*, C_18:1_ ω7*c*, C_17:0_, and C_12:0_ 3-OH. The polar lipid profile was characterized by the presence of phosphatidylethanolamine, phosphatidylglycerol, two unidentified amino lipids, and three unidentified lipids. The major respiratory quinone was Q-8. The finished genome of the strain 16-SW-7 (GenBank assembly accession number: GCA_005877035.1) has a size of 4,531,445 bp and comprises two circular chromosomes L1 and S1, deposited in the GenBank under the accession numbers CP040558 and CP040559, respectively. The strain 16-SW-7 has the ANI values of 98.2% with KMM 638^T^ and KMM 3548^T^ and the DDH values of 84.4 and 83.5%, respectively, indicating clearly that the three strains belonged to a single species. According to phylogenetic evidence and similarity for the chemotaxonomic and genotypic properties, the strain 16-SW-7 (= KCTC 52772 = KMM 701) represents a novel member of the species *Pseudoalteromonas distincta*. Also, we have proposed to reclassify *Pseudoalteromonas paragorgicola* as a later heterotypic synonym of *P. distincta* based on the rules of priority with the emendation of the species.

## Introduction

The genus *Pseudoalteromonas* was proposed by Gauthier et al. (1995) by splitting the genus *Alteromonas* into two genera due to its high level of heterogeneity. At the time of writing, the genus *Pseudoalteromonas* comprises 49 validly published species, including *Pseudoalteromonas haloplanktis* as the type species. Cells of the genus were described as Gram-stain-negative, aerobic, chemoorganotrophic, non-spore-forming, straight and curved rods or ovoid, those are motile by means of a single polar flagellum. All species of the genus were oxidase-positive, required a seawater base for growth, and did not accumulate poly-p-hydroxybutyrate. Subsequently, the genus was emended due to the newly obtained data, including the presence of polar, bipolar, or lateral flagella, gelatin and Tween 80 hydrolysis, glucose fermentation, and the ability of some strains to produce buds and prosthecae (Ivanova et al., 2002a; Hwang et al., 2016; Beurmann et al., 2017). In addition, the G+C content of DNA was extended up to 37–55 mol% (Park et al., 2016). Currently, the G+C content of the genomic DNA ranges from 34.8 mol% for *Pseudoalteromonas denitrificans* DSM 6059^T^ (NCBI RefSeq: NZ_FOLO00000000.1) to 54.9 mol% for *Pseudoalteromonas aestuariivivens* DB-2^T^ (Park et al., 2016). Members of the genus *Pseudoalteromonas* are often isolated from different marine environments, including surface and deep seawater, sediments and sea ice samples, ascidians, coral, a surface slime of a puffer fish, mussels, brown and green algae, diatoms, and the halophyte plants (Bowman, 1998; Sawabe et al., 2000; Egan et al., 2001; Ivanova et al., 2002b,c, 2004; Romanenko et al., 2003a,b; Park et al., 2005; Matsuyama et al., 2013; Wu et al., 2017; Navarro-Torre et al., 2020). In this study, we characterized a non-pigmented strain 16-SW-7, isolated from seawater of the Okhotsk Sea. This strain is attracting the attention of researchers for its ability to convert B red blood cells into the universal type of blood cells (Bakunina et al., 1998; Balabanova et al., 2010). A preliminary study of the taxonomic position of the strain by Sanger sequencing of the 16S rRNA gene indicated its closest relationship with the type strains of the recognized species of the genus *Pseudoalteromonas*, *P. distincta*, and *P. paragorgicola*, with a similarity of 99.9% by the taxonomic EzBioCloud 16S rRNA database (Yoon et al., 2017). The type and single strain of *P. distincta* KMM 638^T^ (formerly *Alteromonas distincta*) was originally isolated from a marine sponge collected at a depth of 350 m near the Komandorskie Islands, Russia (Romanenko et al., 1995). This strain formed non-pigmented colonies and was shown to produce the dark-gray diffusible melanin-like pigments. The type of strain of *P. paragorgicola* KMM 3548^T^ was originally isolated from a gorgonian, *Paragorgia arborea*, collected from the Pacific Ocean, and formed lightly orange-pigmented colonies (Ivanova et al., 2002a). Further investigation presented here on the whole-genome sequences has shown the affiliation of the strain 16-SW-7 and the above-mentioned species with validly published names to the single species. In the present study, we clarify the taxonomic position of the strain 16-SW-7, reclassify the species *P. paragorgicola* as a later heterotypic synonym of *P. distincta*, and specify the description of the species *P. distincta* based on the results of phylogenetic analysis, and genotypic and phenotypic characterization.

## Materials and Methods

### Strain Isolation and Cultivation

The strain 16-SW-7 was isolated from a seawater sample collected near Island Paramushir (Kuril Islands), the Okhotsk Sea, during the 16th cruise of the Research Vessel Academician Oparin by plating 0.1 ml of seawater directly onto nutrient medium as described previously (Nedashkovskaya et al., 2007). After primary isolation and purification, the bacterium was cultivated at 28°C on the same medium or marine agar 2216 (Difco, bioMérieux, Pacific, Biosciences) and stored at −80°C in artificial seawater or marine broth (Difco, bioMérieux, Pacific, Biosciences) supplemented with 20% (v/v) glycerol. The strain 16-SW-7 was deposited in the collection of marine microorganisms (KMM) at the G.B. Elyakov Pacific Institute of Bioorganic Chemistry FEB RAS (Vladivostok, Russia), Korean Collection for the type cultures (KCTC) and VKM under deposit numbers KMM 701, KCTC 52772, and VKM B-2135 D, respectively. The type strains *P. distincta* KMM 638^T^ (=ATCC 700518^T^) and *P. paragorgicola* KMM 3548^T^ (=DSM 26439^T^) were obtained from the collection of marine microorganisms (KMM) and used as the reference strains for comparative taxonomic analysis.

### Morphological, Biochemical, and Physiological Characterization

The physiological, morphological, and biochemical properties of the strain 16-SW-7 were studied using the standard methods. The novel isolate was also examined in the API 20E, API 20NE, API 50 CH, API 32 ID GN, and API ZYM galleries (bioMérieux, France) according to the manufacturer’s instructions, except that the inoculum was prepared using ASW (Bruns et al., 2001) and the galleries were incubated at 28°C. Gram-staining was performed as recommended by Gerhardt et al. (1994). Oxidative or fermentative utilization of glucose was determined on Hugh and Leifson’s medium modified for marine bacteria (Lemos et al., 1985). Catalase activity was tested by the addition of 3% (v/v) H_2_O_2_ solution to a bacterial colony and observation for the appearance of gas. Oxidase activity was determined by using tetramethyl-*p*-phenylenediamine. Degradation of agar, starch, casein, gelatin, chitin, DNA and urea and production of acid from carbohydrates, hydrolysis of Tween 80, nitrate reduction, production of hydrogen sulfide, acetoin (Voges–Proskauer reaction), and indole were tested according to standard methods (Gerhardt et al., 1994). The temperature range for growth was assessed on MA. Tolerance to NaCl was assessed in medium containing 5 g Bacto Peptone (Difco), 2 g Bacto Yeast Extract (Difco), 1 g glucose, 0.02 g KH_2_PO_4_, and 0.05 g MgSO_4_.7H_2_O per liter of distilled water with 0, 0.5, 1.0, 1.5, 2.0, 2.5, 3, 4, 5, 6, 8, 10, 12. 15, 17, 19, and 20% (w/v) of NaCl. Susceptibility to antibiotics was examined on MA plates at 28°C by the routine disk diffusion plate method. Disks were impregnated with the following antibiotics: ampicillin (10 μg), benzylpenicillin (10U), carbenicillin (100 μg), cefalexin (30 μg), cefazolin (30 μg), chloramphenicol (30 μg), erythromycin (15 μg), gentamicin (10 μg), kanamycin (30 μg), lincomycin (15 μg), nalidixic acid (30 μg), neomycin (30 μg), ofloxacin (5 μg), oleandomycin (15 μg), oxacillin (10 μg), polymyxin B (300 U), rifampicin (5 μg), streptomycin (30 μg), tetracycline (5 μg), and vancomycin (30 μg).

### Chemotaxonomic Characterization

For whole-cell fatty acid and polar lipid analysis, the strains 16-SW-7, *P. distincta* KMM 638^T^ and *P. paragorgicola* KMM 3548^T^ were grown under optimal physiological conditions for all strains (at 28°C for 24 h on MA). Cellular fatty acid methyl esters (FAMEs) were prepared according to the methods described by Sasser (1990), using the standard protocol of Sherlock Microbial Identification System (version 6.0, MIDI), and analyzed with the use of a GC-21A chromatograph (Shimadzu) equipped with a fused-silica capillary column (30 m × 0.25 mm) coated with Supelcowax-10 and SPB-5 phases (Supelco) at 210°C. FAMEs were identified by using equivalent chain-length measurements and comparing the retention times to those of authentic standards. The polar lipids of the strains studied were extracted using the chloroform/methanol extraction method of Bligh and Dyer (1959). Two-dimensional TLC of polar lipids was carried out on silica gel 60 F254 (10 cm × 10 cm; Merck) using chloroform/methanol/water (65: 25: 4, by vol.) in the first dimension, and chloroform/methanol/acetic acid/water (80: 12: 15: 4, by vol.) in the second dimension (Collins and Shah, 1984). For detection of the lipids, 10% sulfuric acid in methanol, molybdenum blue, ninhydrin, and *a*-naphthol were applied. Isoprenoid quinones were extracted with chloroform/methanol (2:1, v/v) and purified by TLC, using a mixture of n-hexane and diethyl ether (85:15, v/v) as the solvent. Isoprenoid quinone composition of the strain 16-SW-7 was characterized by HPLC (Shimadzu LC-10A) using a reversed-phase type Supelcosil LC-18 column (15 cm × 4.6 mm) and acetonitrile/2-propanol (65:35, v/v) as a mobile phase at a flow rate of 0.5 ml min^–1^ as described previously (Komagata and Suzuki, 1988).

### Whole-Genome Sequencing and Phylogenetic Analysis

The genomic DNA of the strain *Pseudoalteromonas* sp. 16-SW-7 (=KMM 701 = KCTC 52772) was extracted from the cells grown on marine agar (25°C, 72 h), using a NucleoSpin microbial DNA kit (Macherey-Nagel, 54 Germany), and sequenced at Macrogen, Inc. (Seoul, South Korea). To construct libraries, the high-molecular-weight DNA (15 μg) was fragmented to generate 20-kb SMRTbell™ templates, and then, the fragments were annealed using a PacBio DNA polymerase binding kit and sequenced by the PacBio RS II platform (Pacific Biosciences, United States), with the use of PacBio version 4.0 sequencing kit with single-molecule real-time cells. Hierarchical Genome Assembly Process 3 (HGAP3) was used to perform *de novo* assembly of the PacBio reads for *Pseudoalteromonas* sp. 16-SW-7. The circular shape of the contigs was formed by testing the overlap ability of the contig ends. Because of the mapping reads against the assembled contigs and error correction using Quiver, the final sequence with the highest quality was generated.

The 16S rRNA gene and genome phylogenetic analyses were performed by the Type (Strain) Genome Server (TYGS), an automated high-throughput platform for state-of-the-art genome-based taxonomy (Meier-Kolthoff and Göker, 2019). The genome of the strain 16-SW-7 (GenBank assembly accession: GCA_005877035.1) was compared against all types of strain genomes available in the TYGS database via the MASH algorithm, a fast approximation of intergenomic relatedness (Ondov et al., 2016), and the 10 types of strains with the smallest MASH distances were chosen. In addition, the set of 10 closely related type strains was determined via the 16S rRNA gene sequences, extracted from the genomes using RNAmmer (Lagesen and Hallin, 2007), and BLASTed (Camacho et al., 2009) against the 16S rRNA gene of each of 14,309 type strains. This was used as a proxy to find the best 50 matching type strains, according to the bit score for the 16-SW-7 genome and to subsequently calculate precise distances using the Genome BLAST Distance Phylogeny approach (GBDP) under the algorithm “coverage” and distance formula *d*_5_ (Meier-Kolthoff et al., 2013). These distances were finally used to determine the top 10 closest genomes of the type strains. For the phylogenomic inference, all pairwise comparisons among the set of genomes were conducted using GBDP and accurate intergenomic distances inferred under the algorithm “trimming” and distance formula *d*_5_. One hundred distance replicates were calculated each. Digital DNA-DNA hybridization (DDH) values and confidence intervals were calculated using the recommended settings of the GGDC 2.1 (Meier-Kolthoff et al., 2013). The resulting intergenomic distances were used to infer a balanced minimum evolution tree with branch support via FastME 2.1.6.1 including SPR post-processing (Lefort et al., 2015). Branch support was inferred from 100 pseudo-bootstrap replicates each. The trees were rooted at the midpoint (Farris, 1972) and visualized with PhyD3 (Kreft et al., 2017). The type-based species clustering, using a 70% dDDH radius around each of the 13 type strains, was done as previously described (Meier-Kolthoff and Göker, 2019). Subspecies clustering was done using a 79% dDDH threshold as previously introduced (Meier-Kolthoff et al., 2014).

The whole-genome average nucleotide identity (ANI) values were calculated with the use of ChunLab’s ANI calculator (Yoon et al., 2017). The average amino acid identity (AAI) values were calculated by an AAI-profiler available at http://ekhidna2.biocenter.helsinki.fi/AAI (Medlar et al., 2018). Biosynthetic gene clusters (BGCs) were identified with antiSMASH version 5.1.1 (Medema et al., 2011). The whole-genome sequence analyses and comparative genomics of *Pseudoalteromonas* sp. 16-SW-7, *P. paragorgicola* KMM 3548^T^ (GenBank assembly accession: GCA_014918315.1), and *P. distincta* ATCC 700518^T^ (GenBank assembly accession: GCA_000814675.1) were additionally carried out using the high-performance computing servers Rapid Annotation of microbial genomes using Subsystems Technology (RAST; Overbeek et al., 2014), EzBioCloud (Yoon et al., 2017), and Integrated Microbial Genomes and Microbiomes (IMG/M) system (Chen et al., 2021).

## Results and Discussion

### Morphological, Biochemical, and Physiological Characterization

The strain 16-SW-7 was shown to be a strictly aerobic, heterotrophic, Gram-stain-negative, and motile bacterium, which formed non-pigmented colonies on marine agar and required NaCl or seawater for growth. It was positive for cytochrome oxidase and catalase and hydrolyzed aesculin, casein, gelatin, Tweens 20, 40, and 80, DNA, and tyrosine (Table 1).

**TABLE 1:** Phenotypic characteristics of the strain 16-SW-7 and the closest relatives of the genus *Pseudoalteromonas*.

Characteristic	1	2	3
Source of isolation	Seawater	Sponge	Gorgonian
Colony color	Whitish	Whitish	Pale orange
Flagellation	P	P, L	P
Temperature range for growth (°C)	4–34	4–30	4–30
Salinity range for growth (% NaCl, optimum)	1–10 (2–6)	1–6 (1.5–3)	0.5–8 (1–8)
Requirement for seawater or artificial seawater for growth	−	+	−
Production of melanin-like pigments	−	+	−
H_2_S production	−	+	+
Hydrolysis of:			
Casein	w	−	w
Starch	−	−	+
Alginate	+	+	ND
Acid production from:			
D-Cellobiose, D-galactose, D-glucose, D-lactose, maltose, D-xylose	+	−	+
D-Raffinose	+	−	−
D-Mannitol	+	+	−
Utilization of citrate	+	−	w
Assimilation of (API ID 32GN gallery):			
Itaconic acid, potassium-2-keto-gluconate, L-histidine	+	−	−
D-Melibiose, D-sorbitol	+	−	+
Inositol, sodium malonate, lactic acid, D-ribose, 3-hydroxybutyric acid	−	+	−
Salicin	+	−	−
Enzyme activity (API ZYM tests):			
Cysteine arylamidase, trypsin, α-chymotrypsin	+	−	−
Esterase (C4)	+	+	−
Susceptibility to:			
Ampicillin, vancomycin	−	+	+
Oleandomycin	+	−	+
Tetracycline	−	−	+
DNA G+C content (mol %)	39.3	39.2 (43.8)	39.2

*Strains: 1, 16-SW-7; 2, P. distincta KMM 638^T^; 3, P. paragorgicola KMM 3548^T^. All strains were positive for the following tests: respiratory type of metabolism; motility; presence of oxidase, catalase, alkaline phosphatase, esterase lipase (C8), leucine arylamidase, valine arylamidase, acid phosphatase, and naphthol-AS-BI-phosphohydrolase activities; hydrolysis of aesculin, gelatin, Tweens 20, 40, and 80, DNA, and tyrosine; acid production from sucrose; assimilation of D-glucose, maltose, sucrose, D-mannitol, sodium acetate, L-alanine, L-serine, L-proline, glycogen, propionic acid, valeric acid, and capric acids; susceptibility to carbenicillin, chloramphenicol, erythromycin, doxycycline, gentamicin, kanamycin, nalidixic acid, neomycin, ofloxacin, polymyxin, rifampicin, and streptomycin, and resistance to benzylpenicillin, cefalexin, cefazolin, lincomycin, and oxacillin. All strains were negative for the following tests: nitrate reduction; hydrolysis of agar, chitin, CM-cellulose, and urea; acetoin production; acid production from L-arabinose, D-fructose, D-mannose, D-melibiose, L-rhamnose, D-ribose, D-trehalose, N-acetylglucosamine, and glycerol; assimilation of L-arabinose, L-fucose, L-rhamnose, N-acetylglucosamine, suberic acid, potassium 5-ketogluconate, 3-hydroxybenzoic acid, 4-hydroxybenzoic acid, and salicin; lipase (C14), α-galactosidase, β-galactosidase, β-glucuronidase, α-glucosidase, β-glucosidase, N-acetyl-β-glucosaminidase, α-mannosidase, and α-fucosidase activities. P, polar; L, lateral; +, positive; −, negative; w, weak reaction; ND, no data available.*

The strains 16-SW-7, *P. distincta* KMM 638^T^, and *P. paragorgicola* KMM 3548^T^ shared many common phenotypic features, such as respiratory type of metabolism, motility by means of flagella, the presence of catalase, alkaline phosphatase, esterase lipase (C8), leucine arylamidase, valine arylamidase, acid phosphatase, and naphthol-AS-BI-phosphohydrolase activities (Table 1). They could not synthesize lipase (C14), *N*-acetyl-β-glucosaminidase, β-glucosidase, α-galactosidase, β-glucuronidase, α-mannosidase and α-fucosidase, hydrolyse agar, chitin, and urea and reduce nitrate to nitrite. However, the strain 16-SW-7 can be distinguished from its closest phylogenetic relatives by the several phenotypic traits, including the ability to form acid from D-raffinose, to produce hydrogen sulfide and cysteine arylamidase, trypsin and α-chymotrypsin, and to be resistant to ampicillin and vancomycin (Table 1). The above findings can extend the phenotypic characteristics those were reported for the species *P. distincta* (Romanenko et al., 1995; Ivanova et al., 2000, 2004) after justification of the placement of the strains 16-SW-7 and *P. paragorgicola* KMM 3548^T^ in the species *P. distincta*.

### Chemotaxonomic Characterization

The fatty acid profiles of the strains 16-SW-7, *P. distincta* KMM 638^T^, and *P. paragorgicola* KMM 3548^T^ were similar (Table 2). The predominant fatty acids (>5% of the total fatty acids) of the strain 16-SW-7 and its closest relatives were C_16:1_ ω7*c* (29–32.1%), C_16:0_ (15.4–18.2%), C_17:1_ ω8*c* (11.7–17.9%), C_18:1_ ω7*c* (5.2–11%), C_17:0_ (5.8–10.3%), and C_12:0_ 3-OH (4.8–7.5%). The composition of other fatty acids presented in Table 2 was also similar except that the strain 16-SW-7 contained higher proportions of C_14:0_, C_12:0_, and iso-C_16:0_, and lower proportions of C_15:1_ ω8c, C_12:0_ 3-OH, and C_13:0_ 3-OH. These values were consistent with the results of phylogenetic analysis and confirmed the affiliation of the strains studied to the same species. The polar lipid profile of the strain 16-SW-7 was characterized by the presence of phosphatidylethanolamine, phosphatidylglycerol, two unidentified amino lipids, and three unidentified lipids (Table 2 and Supplementary Figure 1). It was similar to that of *P. paragorgicola* KMM 3548^T^ and it can be distinguished from another relative, *P. distincta* KMM 638^T^, by the presence of unknown lipids L1 and L2. The nearest neighbors of the strains under study, *Pseudoalteromonas aliena* LMG 22059^T^ and *Pseudoalteromonas fuliginea* KMM 216^T^, distinguished from them by the presence of unknown phospholipids and unknown aminophospholipid and two unknown glycolipids, respectively (Machado et al., 2016; Zhang et al., 2016). The main respiratory quinone of the strains under study was ubiquinone Q-8 that is consistent with those reported for the members of the family *Pseudoalteromonadaceae* (Ivanova et al., 2004).

**TABLE 2:** Fatty acid composition (%) of the strain 16-SW-7 and closely related strains of the genus *Pseudoalteromonas.*

Fatty acids	1	2	3
**Saturated**			
C_12:0_	2.8	tr	tr
C_14:0_	3.9	tr	1.3
C_15:0_	2.5	4.7	3.0
**C_16:0_**	**18.2**	**15.4**	**16.0**
**C_17:0_**	**6.1**	**10.3**	**5.8**
C_18:0_	1.9	1.8	1.4
**Unsaturated**			
C_15:1_ ω*8c*	2.7	4.3	4.9
**C_16:1_ ω*7c***	**30.6**	**29.0**	**32.1**
**C_17:1_ ω8c**	**11.7**	**17.9**	**15.3**
**C_18:1_ ω7c**	**11.0**	**5.2**	**7.5**
**Branched**			
iso-C_16:0_	1.0	tr	tr
**Hydroxy**			
**C_12:0_ 3-OH**	**4.8**	**6.3**	**7.5**
C_13:0_ 3-OH	tr	1.2	1.0

*Strains: 1, 16-SW-7; 2, P. distincta KMM 638^T^; 3, P. paragorgicola KMM 3548^T^. All data are from the present study. Major components (≥5.0%) are highlighted in bold. tr, trace amount (<1.0%).*

### 16S rRNA Genes and Phylogenomic Analysis

The analysis of the 16S rRNA gene sequence of the strain 16-SW-7 (GenBank accession number: OL587468) in the EzTaxon database application (Yoon et al., 2017) revealed 100% similarity with *Pseudoalteromonas arctica* A 37-1-2^T^ (CP011026) and *Pseudoalteromonas elyakovii* KMM 162^T^ (AF082562), and 99.9% similarity with *P. distincta* KMM 638^T^ (JWIG01000030) and *P. paragorgicola* KMM 3548^T^ (AY040229). However, the 16S rRNA gene sequences obtained by the Sanger method are recommended to be compared with the genome sequences, as well as the use of overall genome data for the taxonomy of prokaryotes, such as average nucleotide identity (ANI) and digital DDH (dDDH) and relatedness between the strains and type of strain of a species (Chun et al., 2018). The closed genome of 16-SW-7 was found to contain nine full-length sequences of 16S rRNA genes with 99.87–100% similarity between each other (Table 3). The multiple 16S rRNA genes seem to be a characteristic of the type species of the family *Pseudoalteromonadaceae*, including *P. distincta* KMM 638^T^ and *P. paragorgicola* KMM 3548^T^ (Table 3). Among the nine 16S rRNA genes found in the genome of *P. distincta* KMM 638^T^ (=ATCC 700518^T^), only one—the length-comparable gene in a contig 30—was extracted for the analysis, probably due to an incomplete genome sequencing (GenBank WGS accession: JWIG00000000.1). In the *P. paragorgicola* KMM 3548^T^ genome, two 16S rRNA genes were completely sequenced among three found (GenBank WGS accession: AQHE00000000.1).

**TABLE 3:** The 16S rRNA gene sequences content and similarity for the strains *Pseudoalteromonas* sp. 16-SW-7, *P. distincta* ATCC 700518^T^, and *P. paragorgicola* KMM 3548^T^.

IMG homolog*	NCBI homolog genome locus_tag (16S rRNA gene)	Identity %	Identity/length	Genome ID	Genome name	Contig/length	Coordinates/strand
2888223316	FFU37_04590 (OL587469)	100.00	1536/1536	CP040558	*Pseudoalteromonas* sp. 16-SW-7	1(L1)/3735685	1023946..1025481/+
2888222433	FFU37_00210 (OL587468)	100.00	1536/1536	CP040558	*Pseudoalteromonas* sp. 16-SW-7	1(L1)/3735685	48439..49974/+
2888226108	FFU37_18380 (OL587475)	99.94	1535/1536	CP040559	*Pseudoalteromonas* sp. 16-SW-7	2(S1)/795760	375599..377134/+
2888225489	FFU37_15325 (OL587473)	99.94	1535/1536	CP040558	*Pseudoalteromonas* sp. 16-SW-7	1(L1)/3735685	3416198..3417733/−
2888225372	FFU37_14755 (OL587472)	99.94	1535/1536	CP040558	*Pseudoalteromonas* sp. 16-SW-7	1(L1)/3735685	3277653..3279188/−
2888225547	FFU37_15610 (OL587474)	99.87	1534/1536	CP040558	*Pseudoalteromonas* sp. 16-SW-7	1(L1)/3735685	3477632..3479167/+
2888225264	FFU37_14215 (OL587471)	99.87	1534/1536	CP040558	*Pseudoalteromonas* sp. 16-SW-7	1(L1)/3735685	3172769..3174304/−
2888222403	FFU37_00060 (OL587467)	99.87	1534/1536	CP040558	*Pseudoalteromonas* sp. 16-SW-7	1(L1)/3735685	16934..18469/+
2888224778	FFU37_11820 (OL587470)	99.74	1533/1536	CP040558	*Pseudoalteromonas* sp. 16-SW-7	1(L1)/3735685	2652584..2654119/−
–	QT16_19995	99.94	1542/1543	JWIG01000030	*P. distincta* strain ATCC 700518^T^	C30/180150	175393..176935/+
–	PPAR_aR004	99.87	1522/1524	AQHE01000014	*P. paragorgicola* KMM 3548^T^	14/767276	187495..189018/+
–	PPAR_aR007	100.00	1524/1524	AQHE01000021	*P. paragorgicola* KMM 3548^T^	21/300025	298305..299828/+

*From the alignment on query gene of the strain 16-SW-7 under the IMG/M database accession number 2888222433 (FFU37_00210/OL587468), implemented by Top IMG Isolate RNA hits or NCBI BLAST to get top RNA homologs.

**FIGURE 1:**
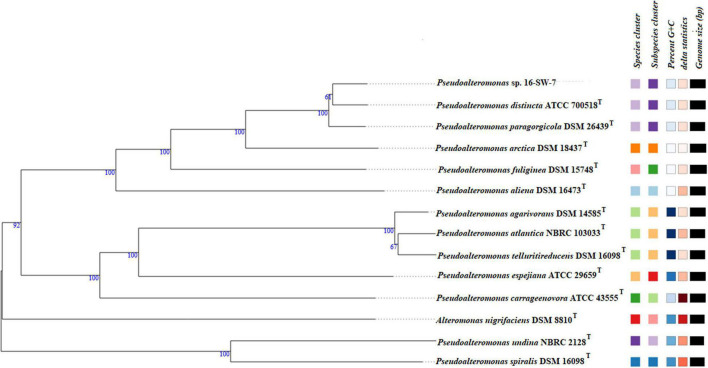
Tree inferred with FastME 2.1.6.1 from GBDP distances calculated from genome sequences by the TYGS server (Lefort et al., 2015). The branch lengths are scaled in terms of GBDP distance formula d5. The numbers above the branches are GBDP pseudo-bootstrap support values >60% from 100 replications, with an average branch support of 92.7%. The tree was rooted at the midpoint (Farris, 1972). The square labels colored by the same color indicate the genomes with the same species (light lilac) and subspecies (dark lilac) gene clusters, G+C content (white), and delta statistics: *Pseudoalteromonas*_GCF_005877035.1 (the strain 16-SW-7), *P. distincta* ATCC 700518^T^, and *P. paragorgicola* DSM 26439^T^ form one phylogenomic clade separated from other species of the genus.

The whole-genome sequence of the strain 16-SW-7 was 4,531,445 bp, with a G+C content of 39.3 mol% and comprised of two circular chromosomes L1 and S1, deposited in the GenBank under the accession numbers CP040558 and CP040559, respectively (assembly accession: GCA_005877035.1). The genome size of *P. paragorgicola* KMM 3548^T^ (GenBank assembly accession: GCA_014918315.1) was 4,322,351 bp, with the G+C content 39.2 mol%. In comparison, the genome size of *P. distincta* KMM 638^T^ (GenBank assembly accession: GCA_000814675.1) was 4,532,748 bp and the G+C content of 39.2 mol% (Supplementary Table 1). The GBDP phylogenomic tree is consistent with the branching patterns, observed for only the strains 16-SW-7 and *P. paragorgicola* KMM 3548^T^ (=DSM 26439^T^) in the 16S rRNA gene sequence-based tree, generated by TYGS, because of the use of the *P. distincta* KMM 638^T^ (=ATCC 700518^T^) gene with lower identity (Table 3, Figure 1, and Supplementary Figure 2).

However, the species-specific gene clusters for the strains 16-SW-7, *P. paragorgicola* KMM 3548^T^ (=DSM 26439^T^), and *P. distincta* KMM 638^T^ (=ATCC 700518^T^) were identical, indicating that the three strains belonged to a single species (Figure 1 and Supplementary Figure 2). The ANI calculator in EzBioCloud, based on the use of OrthoANIu algorithm (Lee et al., 2016), showed the similar high ANI values (98.04–98.2%) for the strain 16-SW-7 and the reference strains *P. distincta* KMM 638^T^ and *P. paragorgicola* KMM 3548^T^ (Supplementary Table 2), which are higher than the species-level cutoff value of 95–96% (Richter and Rosselló-Móra, 2009). According to the TYGS results, the dDDH (d4) values between the 16-SW-7 genome and genomes of *P. distincta* and *P. paragorgicola* were 84.4 and 83.5%, respectively (Supplementary Table 3), that are sufficiently higher than the suggested species boundary of 70% (Wayne et al., 1987). The proteome-wide sequence search results, implemented by a web server AAI-profiler (Medlar et al., 2018), showed 98.6 (87% matched proteins) and 98.8% (85.9% matches) average amino acid identity (AAI) of the strain 16-SW-7 with *P. distincta* and *P. paragorgicola*, which are higher than 85% proposed as a threshold for delimitation of a species (Goris et al., 2007).

The target count for *P. paragorgicola* proteins showed 98.8 and 98.5% AAI (83.5 and 83.1% matches) against the proteins of *Pseudoalteromonas* sp. 16-SW-7 and *P. distincta*, respectively. The calculated genetic similarities are presented in Supplementary Tables 1–3. These values support the proposed affiliation of the three strains to a single species of the genus *Pseudoalteromonas*.

### Genome Features and Comparative Genomics

The core-gene content (about 3200 genes according to EzBioCloud) and coding sequences (CDSs) similarity confirmed the affiliation of the strains 16-SW-7 and *P. paragorgicola* KMM 3548^T^ to the species *P. distincta* (Supplementary Figures 3, 4 and Supplementary Tables 4–6). They include genes for use of some common mechanisms to respond to high salt stress, such as a high-affinity choline and betaine uptake system (Supplementary Table 4, Column 1: lines 769, 898, 1257, 2699, 3969–3974), the doubled genes for the glutamate synthase small and large subunits (Supplementary Table 4, Column 1: lines 1761, 3562, 2003, 2033), K^+^ transporters Trk H/A (Supplementary Table 4, Column 1: lines 3064, 3069), other ABC transporters/permeases, and transcription factors (Fu et al., 2014). However, analysis of the subsystem features, annotated by RAST, indicated the presence of some individual functions for the strains 16-SW-7, *P. distincta* KMM 638^T^ (=ATCC 700518^T^), and *P. paragorgicola* KMM 3548^T^ (Table 4).

**TABLE 4:** Genome-based comparison for the presence/absence of metabolic subsystem functions in *Pseudoalteromonas* sp. 16-SW-7 (A), *P. distincta* ATCC 700518^T^ (B), and *P. paragorgicola* KMM 3548^T^ (C).

Category	Subcategory	Subsystem	Function	A	B	C
Amino acids and derivatives	Lysine, threonine, methionine, and cysteine	Methionine biosynthesis (MB)	Homoserine O-acetyltransferase (EC 2.3.1.31), MB subpathway	+	−	−
Amino acids and derivatives; protein metabolism	Arginine; urea cycle, polyamines	Urea decomposition; G3E family of P-loop GTPases (metallocenter biosynthesis)	Urea ABC transporter, ATPase protein UrtD, UrtE, UrtB, UrtC; Urease accessory proteins UreD, UreE, UreF, UreG; Urease alpha, beta, and gamma subunits (EC 3.5.1.5)	−	+	−
Carbohydrates	Monosaccharides	Mannose Metabolism	GDP-mannose mannosyl hydrolase (EC 3.6.1.), Phosphomannomutase (EC 5.4.2.8)	+	−	−
Cell wall and capsule	Capsular and extracellular polysaccharides	Rhamnose containing glycans; dTDP-rhamnose synthesis	Alpha-1,2(1,3)-L-rhamnosyltransferase (EC 2.4.1.); polysialic acid transporter KpsM; dTDP-4-dehydrorhamnose 3,5-epimerase (EC 5.1.3.13) and reductase (EC 1.1.1.133); dTDP-rhamnosyltransferase RfbF	−	+	−
DNA metabolism	DNA repair	DNA repair, bacterial RecBCD pathway	RecD-like DNA helicase Atu2026 (exodeoxyribonuclease V)	+	−	−
DNA metabolism	DNA repair	DNA repair, bacterial	DNA-cytosine methyltransferase (EC 2.1.1.37), modulates gene expression, a component of bacterial restriction-modification systems	+	−	−
DNA metabolism	No subcategory	Restriction-modification system	Type III restriction-modification system methylation and helicase subunits (EC 2.1.1.72), host-protective DNA methylation	−	−	+
DNA metabolism	No subcategory	Restriction-modification system	Putative DNA-binding protein in cluster with Type I restriction-modification system	−	+	−
Fatty acids, lipids, and isoprenoids	No subcategory	Polyhydroxybutyrate metabolism	D-beta-hydroxybutyrate permease (utilization of poly-HB, gluconate)	+	−	−
Membrane transport	Protein secretion system, Type II	Widespread colonization island	Flp pilus assembly protein, pilin Flp	***−***	***−***	+
Membrane transport	TRAP transporters	TRAP transporter collection	TRAP-type C4-dicarboxylate transport system, uptake host’s succinate, fumarate, and malate during symbiotic growth	−	−	+
Membrane transport; virulence and defense	Cation transporters; resistance to toxic compounds	Copper transport system; Cu^2+^ homeostasis	Copper resistance proteins CopC, CopD, CopB; multicopper oxidase	−	+	−
Nucleosides and nucleotides	Detoxification	Housecleaning nucleoside triphosphate pyrophosphatases	Deoxyuridine 5′-triphosphate nucleotidohydrolase (EC 3.6.1.23), remove dUTP for preservation of genetic integrity for growth and virulence	−	−	+
Regulation and cell signaling	Programmed cell death and toxin-antitoxin systems	Phd-Doc, YdcE-YdcD toxin-antitoxin (programmed cell death) systems	Death on curing protein, doc toxin [mimicker of aminoglycoside antibiotic hygromycin B (HygB), increase in mRNA half-life]	−	−	+
Regulation and cell signaling	No subcategory	DNA-binding regulatory proteins, strays	Aromatic hydrocarbon utilization transcriptional regulator CatR (LysR family)	−	−	+
Regulation and cell signaling	No subcategory	Orphan regulatory proteins	Sensor kinase CitA, DpiB (EC 2.7.3.-), involved in anaerobic citrate catabolism in response to anaerobic conditions	−	+	−
Stress response	Cold shock	Cold shock, CspA family of proteins	Cold shock protein CspD	−	−	+
Stress response	Osmotic stress	Choline and betaine uptake and betaine biosynthesis	GbcA glycine betaine demethylase, transporter OpuD [utilization of quaternary ammonium compounds at high osmolalities (kidneys)]	−	+	−
RNA metabolism	RNA processing and modification	Ribonucleases in bacillus	Metallo-beta-lactamase family protein, RNA-specific	−	+	−

The RAST comparative genomics revealed 242 and 426 strain-specific CDSs (singletons) in the strain 16-SW-7 against the strains KMM 638^T^ (=ATCC 700518^T^) and KMM 3548^T^, respectively, most of which encoded hypothetical proteins, and only 40 and 50 genes, respectively, had a function (Supplementary Table 4). Meanwhile, 1378 and 1229 CDSs of KMM 638^T^ (=ATCC 700518^T^) and KMM 3548^T^, respectively, had 100% identity with the CDSs of 16-SW-7 (Supplementary Table 5). In the genome of the strain KMM 3548^T^, 1259 identical CDS and 481 singletons were found vs. the strain KMM 638^T^ (ATCC 700518^T^), and 420 singletons vs. the strain 16-SW-7 (Supplementary Table 6). All three strains putatively have xylan and xylose degradation specialization, but differ from each other by exopolysaccharide and lipopolysaccharide biosynthesis pathways (Table 4 and Supplementary Tables 4, 6), which are known to be responsible for serotypes in clinical bacterial strains (Balabanova et al., 2020). Thus, the strain 16-SW-7 exclusively contains the doubled genes encoding for GDP-mannose mannosyl hydrolase, phosphomannomutase, and several sugar and glycosyltransferases, as well as the low-identical capsular polysaccharide synthases (up to 78–79%) of the type strain KMM 638^T^ (ATCC 700518^T^): A, B, C, D, UDP-glucose 4-epimerase, lipid carrier: UDP-N-acetylgalactosaminyltransferase, O-antigen acetylase, N-acetylneuraminate cytidylyltransferase (sialic acid synthesis), polysaccharide deacetylase, dTDP-4-dehydrorhamnose reductase, and specific lipoproteins which are absent in KMM 3548^T^ (Supplementary Tables 4, 6). Meanwhile, KMM 3548^T^ includes many genes for rhamnosyltransferases, some of which are similar only to KMM 638^T^ (ATCC 700518^T^), and related hydrolases, lipoproteins, and capsular polysaccharide synthesis and export systems (Supplementary Table 6). In addition, 16-SW-7 and KMM 638^T^ (ATCC 700518^T^) differ from KMM 3548^T^ by some genes responsible for xanthine and gluconate metabolism, carbon starvation, catechol pathway, and phosphate metabolism (Supplementary Table 4, Column 1: lines 3684–3691). Contrarily, the strain KMM 3548^T^ has almost all genes found in the strains 16-SW-7 and ATCC 700518^T^, but mostly distinguishes from them by the large number of mobile elements, transposases, integrases, chaperone proteins of heat shock (HtpG), components of fatty acid synthases, enlarged pectin degradation system, and hypothetical proteins of unknown function (Supplementary Table 6). In general, the different level of identity and numbers of the genes for motility functions, metal resistance, TonB-related receptors, transporters, beta-lactamases, bactericins, signal transduction (sensors, receptors, transporters, enzymes), mobile elements, and DNA repair systems reflect eco-physiological diversity and different adaptive lifestyles of the free-living 16-SW-7, and host-associated KMM 3548^T^ and KMM 638^T^ (ATCC 700518^T^) (Table 4 and Supplementary Tables 4–6). Indeed, KMM 638^T^ (ATCC 700518^T^) has acquired the additional pathogenic-like systems for osmotic stress response, urea decomposition, and sensor kinase CitA (Table 4), indicating an ability to live in the host tissues with a high osmolality and use citrate fermentation under anaerobic conditions (Wetzel et al., 2011; Scheu et al., 2012). KMM 3548^T^ rather contains rhizobia-like symbiotic traits (Table 4), such as TRAP-type C4-dicarboxylate transport system for facultatively anaerobically fumarate and nitrate respiration (Janausch et al., 2002), aromatic hydrocarbon utilization regulator CatR, Type III restriction-modification system that confers host DNA protection via methylation (Murray et al., 2021). Meanwhile, the free-living strain 16-SW-7 is enforced by the modification and repair systems of its own DNA and methylation-dependent biosynthetic pathways, such as two bacteriocin BGCs (found by antiSMASH), defending it from UV excess, environmental competitors, and facilitating the rapid genetic rearrangement (Table 4).

In addition, the strains KMM 638^T^ (ATCC 700518^T^) and KMM 3548^T^ produce brown and lightly orange pigments, respectively, in comparison with the colorless free-living 16-SW-7 colonies, probably due to its active dye-decolorization peroxidase (Supplementary Table 4, Column 1: 2637) or inactive pigment-producing genes. *P. distincta* KMM 638^T^ (ATCC 700518^T^) was reported to produce diffusible dark-gray-colored melanin-like pigment (pyomelanin) (Romanenko et al., 1995), the responsible genes of which are found in each strain (Supplementary Table 4, column 1: 2123, 3887). For many pathogens, melanin production is associated with virulence and completely suppressed at 30–35°C (Turick et al., 2009). According to the gene content (Supplementary Tables 4–6), *P. distincta* can produce different colored polyketide secondary metabolites from simple fatty acids (pigments, antioxidants, toxins), which increase virulence for many pathogens by improving its intracellular survival (Woo et al., 2012; Hug et al., 2020). Probably, KMM 638^T^ (ATCC 700518^T^) and KMM 3548^T^ developed and fixed their pigment-producing phenotypic traits due to colonizing psychrophilic marine organisms (Romanenko et al., 1995; Ivanova et al., 2002a). Thus, the slightly orange pigments in KMM 3548^T^ could belong to antioxidant xanthomonadin-like pigments (carotenoids, anthraquinones, zeaxanthin, flexirubins), which protect against oxidative stress and UV exposure, establishing or maintaining commensal relationships between bacteria and their hosts (He et al., 2020). At least, the resorcinol and arylpolyene biosynthetic gene clusters (66209 nucleotide region), which are functionally related to antioxidative carotenoids, were found in the KMM 3548^T^ genome by antiSMASH (Medema et al., 2011; Schöner et al., 2016).

In any case, all three genomes of *P. distincta* possess many polyketide synthases and concomitant proteins with a high level of identity (97–100%) (Supplementary Tables 4–6). The dark shade of pigments in KMM 638^T^ (ATCC 700518^T^) may be due to the degree of expansion of the pyomelanin production and weakening xanthine metabolism by some genes lost, compared to the KMM 3548^T^ pathway (Supplementary Table 6).

In conclusion, based on the results of the above phylogenetic, phenotypic, and chemotaxonomic study, we suggest that the strain 16-SW-7 is affiliated to the species *P. distincta*. Moreover, the high similarities in the genomic sequences and phenotypic characteristics found between the species *P. distincta* and *P. paragorgicola* places them in the same species. Therefore, it is proposed to reclassify the species *P. paragorgicola* as a later heterotrophic synonym of *P. distincta* in accordance with the rules of priority of prokaryotic names, governed by the International Code of Nomenclature of Bacteria (Parker et al., 2019), and to emend the description of the species *P. distincta*.

### Emended Description of the Species *Pseudoalteromonas distincta* (Romanenko et al. 1995) Ivanova et al. 2000

The description of the species *Pseudoalteromonas distincta* is as given by Romanenko et al. (1995) and Ivanova et al. (2000, 2002a) with the following modifications and amendments. Cells are Gram-stain-negative, non-spore-forming, strictly aerobic rods, motile by means of a single polar or four to seven lateral flagella. On marine agar, colonies are non-pigmented or slightly orange colored. They can produce diffusible melanin-like pigments. Cells are catalase- and oxidase-positive. They require Na^+^ ions or sea water for growth. Growth occurs in media with 0.5–10% NaCl. Temperature for growth ranges from 4 to 34°C. Aesculin, gelatin, Tweens 20, 40, and 80, DNA, alginate, and tyrosine are hydrolyzed but agar, chitin, CM-cellulose, and urea are not hydrolyzed. Hydrolysis of casein and starch is strain dependent. Acid is formed from sucrose but not from L-arabinose, D-fructose, D-mannose, D-melibiose, L-rhamnose, D-ribose, D-trehalose, *N*-acetylglucosamine, and glycerol. Some strains can produce acid from D-cellobiose, D-galactose, D-glucose, D-lactose, maltose, D-raffinose, D-xylose, and D-mannitol and utilize citrate. In API ID 32GN gallery, they are positive for D-glucose, maltose, sucrose, D-mannitol, sodium acetate, sodium citrate, L-alanine, L-serine, L-proline, glycogen, propionic acid, valeric acid, and capric acids. Assimilation of inositol, sodium malonate, lactic acid, D-ribose, 3-hydroxybutyric acid, itaconic acid, potassium-2-keto-gluconate, L-histidine, and salicin is variable. In API ZYM gallery, alkaline phosphatase, esterase lipase (C8), leucine arylamidase, valine arylamidase, acid phosphatase, and naphthol-AS-BI-phosphohydrolase activities are present but lipase (C14), α-galactosidase, β-galactosidase, β-glucuronidase, α-glucosidase, β-glucosidase, *N*-acetyl-β-glucosaminidase, α-mannosidase, and α-fucosidase activities are absent. Esterase (C4), cysteine arylamidase, trypsin, and α-chymotrypsin can be produced. Nitrate is not reduced to nitrite. Acetoin and indole are not produced. Production of hydrogen sulfide is strain dependent. The predominant fatty acids (>5% of the total fatty acids) were C_16:1_ ω7*c*, C_16:0_, C_17:1_ ω8*c*, C_18:1_ ω7*c*, C_17:0_, and C_12:0_ 3-OH. The polar lipid profile was characterized by the presence of phosphatidylethanolamine, phosphatidylglycerol, two unidentified amino lipids, and three unidentified lipids. The major respiratory quinone is ubiquinone Q-8. The genomic DNA G+C content is 39.2–39.3 mol%. The genome size is 4.3–4.5 Mb. The type of strain is KMM 638^T^ (=ATCC 700518^T^), isolated from a marine sponge collected at a depth of 350 m near the Komandorskie Islands, Russia. The GenBank/EMBL/DDBJ assembly accession number for the genome of the type of strain is GCA_000814675.1.

## Data Availability Statement

The datasets presented in this study can be found in online repositories. The names of the repository/repositories and accession number(s) can be found in the article/Supplementary Material.

## Author Contributions

ON and LB contributed to conception and designed of the study. ON, S-GK, LB, and NZ performed the experimental works. ON, S-GK, LB, NZ, OS, LT, and VM wrote sections of the manuscript. All authors contributed to manuscript revision, read, and approved the submitted version.

## Conflict of Interest

The authors declare that the research was conducted in the absence of any commercial or financial relationships that could be construed as a potential conflict of interest.

## Publisher’s Note

All claims expressed in this article are solely those of the authors and do not necessarily represent those of their affiliated organizations, or those of the publisher, the editors and the reviewers. Any product that may be evaluated in this article, or claim that may be made by its manufacturer, is not guaranteed or endorsed by the publisher.
