# Supplementary material for: Genome-Based Classification of Strain 16-SW-7, a Marine Bacterium Capable of Converting B Red Blood Cells, as Pseudoalteromonas distincta and Proposal to Reclassify Pseudoalteromonas paragorgicola as a Later Heterotypic Synonym of Pseudoalteromonas distincta
Source: Front Microbiol. 2022 Feb 8;12:809431. doi: 10.3389/fmicb.2021.809431 (PMC8865838; doi:10.3389/fmicb.2021.809431)
Supplement: Supplementary file 1 [file Data_Sheet_1.docx]

Supplementary Material

# Supplementary Data

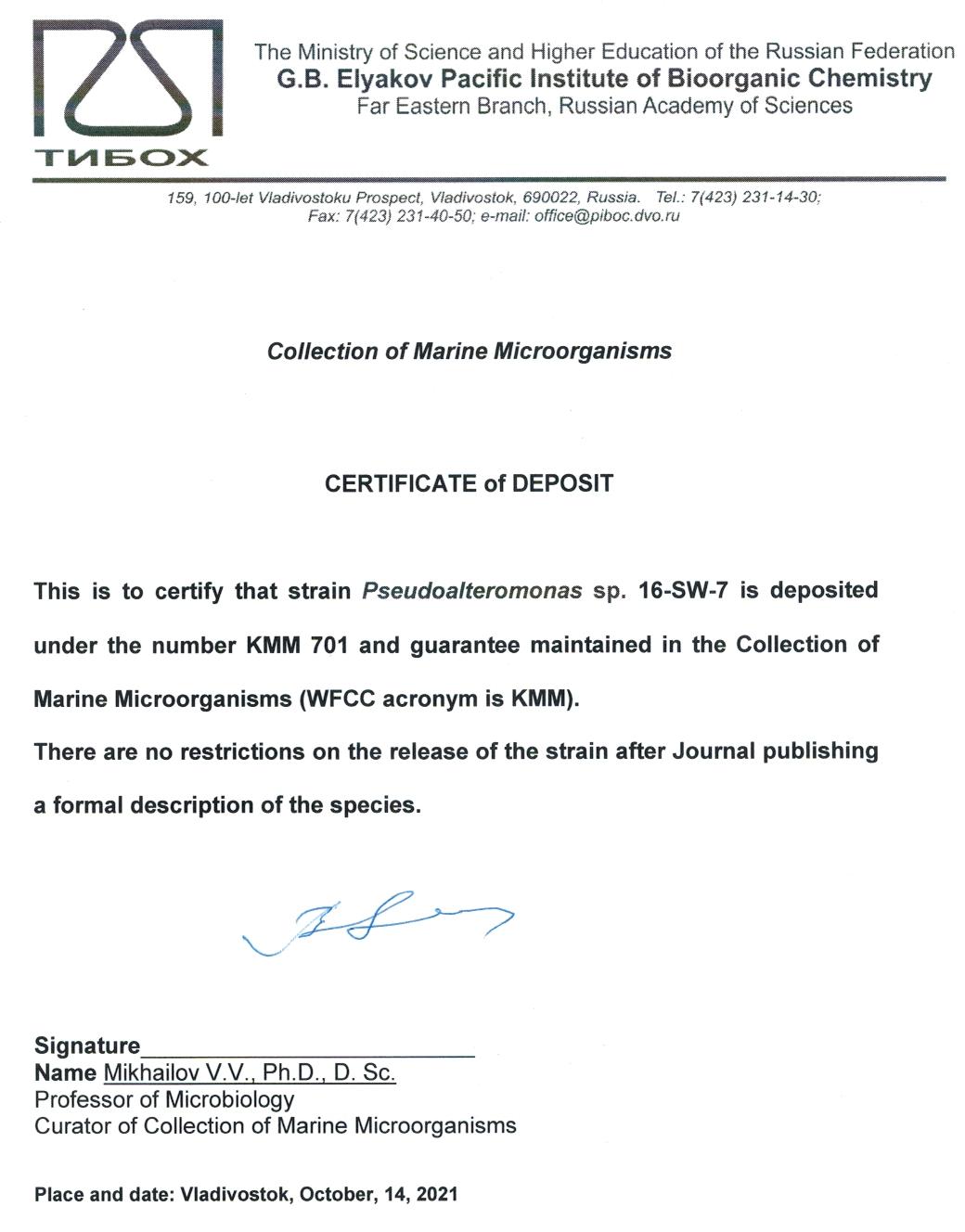


# Supplementary Figures and Tables

## Supplementary Figures

**
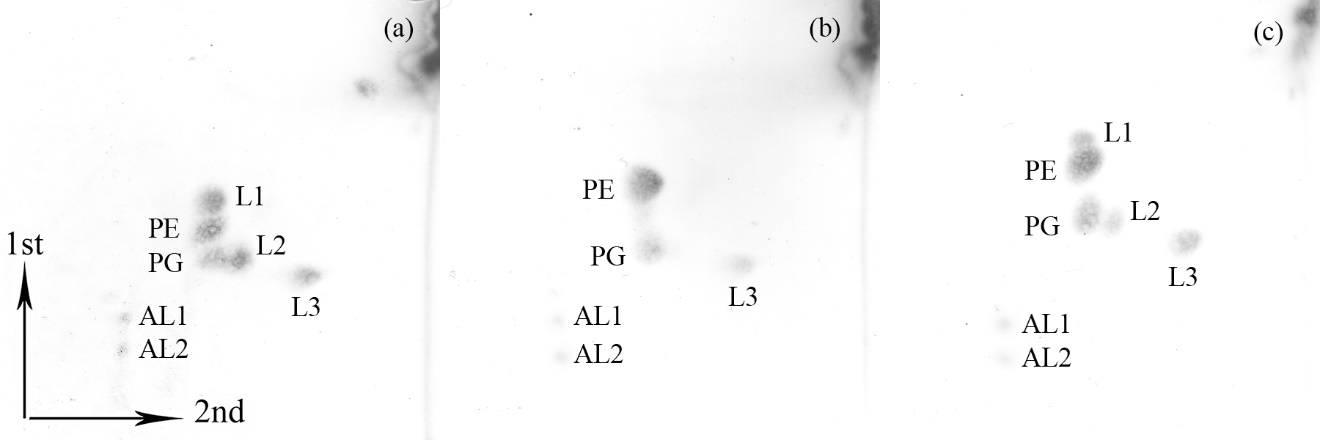
**

**Supplementary Figure 1.** Two-dimensional thin-layer chromatogram of polar lipids extracted from strains 16-SW-7 (a), *Pseudoalteromonas* *distincta* KMM 638^T^ (b) and *Pseudoalteromonas* *paragorgicola* KMM 3548^T^ (c). PE, phosphatidylethanolamine; PG, phosphatidylglycerol, AL1 and AL2, unidentified amino lipids; L1, L2 and L3, unidentified lipids.

**
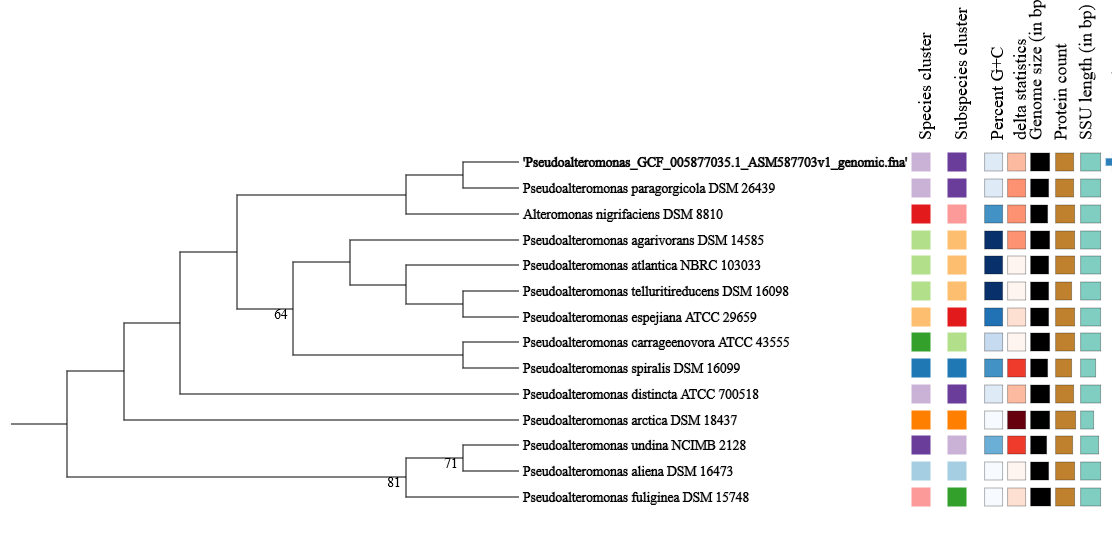
**

## Supplementary Figure 2. Tree inferred by the TYGS server with FastME 2.1.6.1 9 from GBDP distances calculated from 16S rDNA gene sequences (the query ‘Pseudoalteromonas_GCF_005877035.1_ASM587703v1_genomic.fna’ is the genomic 16S rDNA of the strain 16-SW-7). The branch lengths are scaled in terms of GBDP distance formula *d_5_*. The numbers above branches are GBDP pseudo-bootstrap support values > 60 % from 100 replications, with an average branch support of 35.4 %. The tree was rooted at the midpoint. The only type strains are used in TYGS.


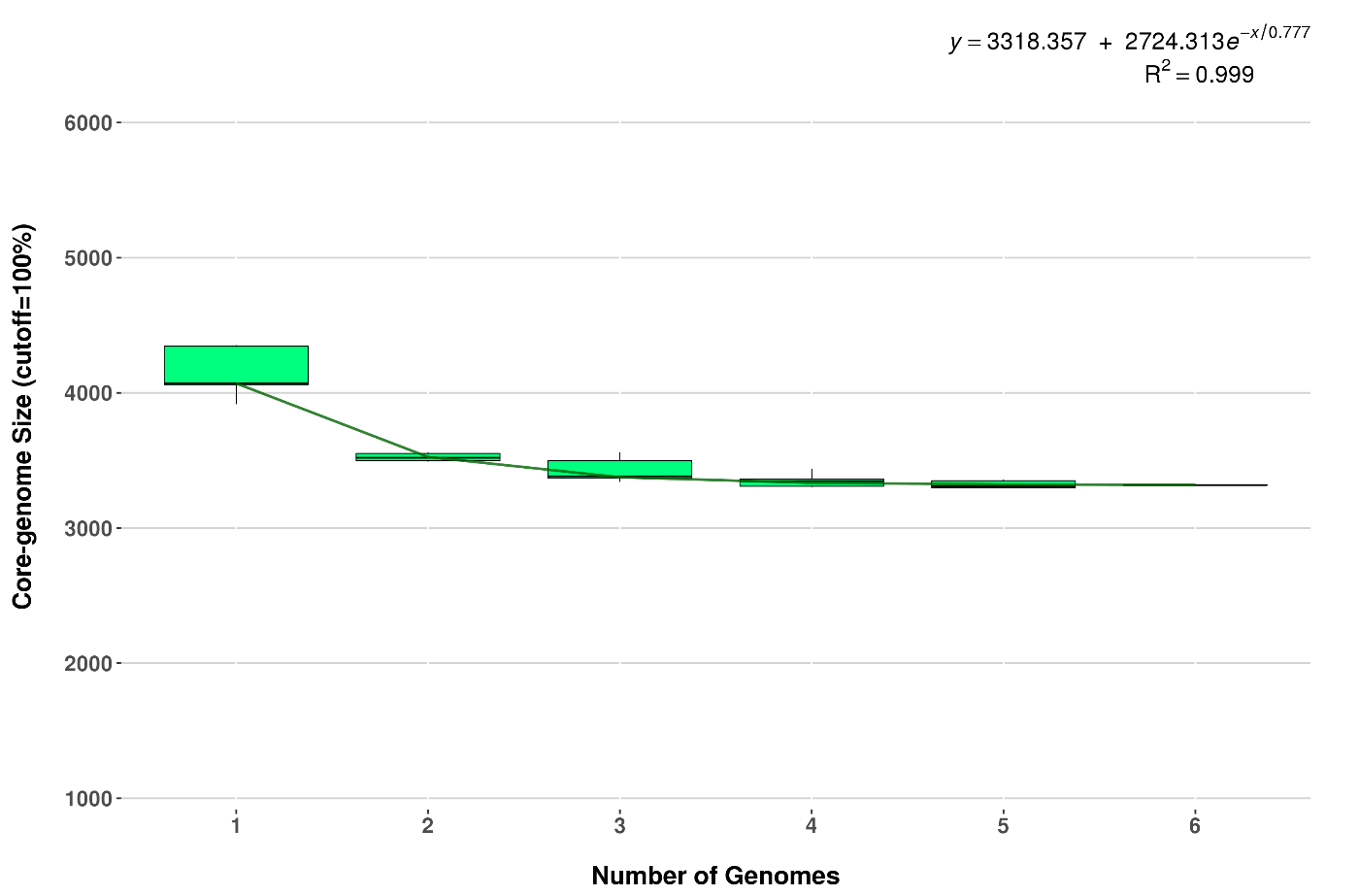


**Supplementary Figure 3.** Core-genome curve for the POG number of the species *Pseudoalteromonas distincta* calculated by EzBioCloud comparative genomics (CG).


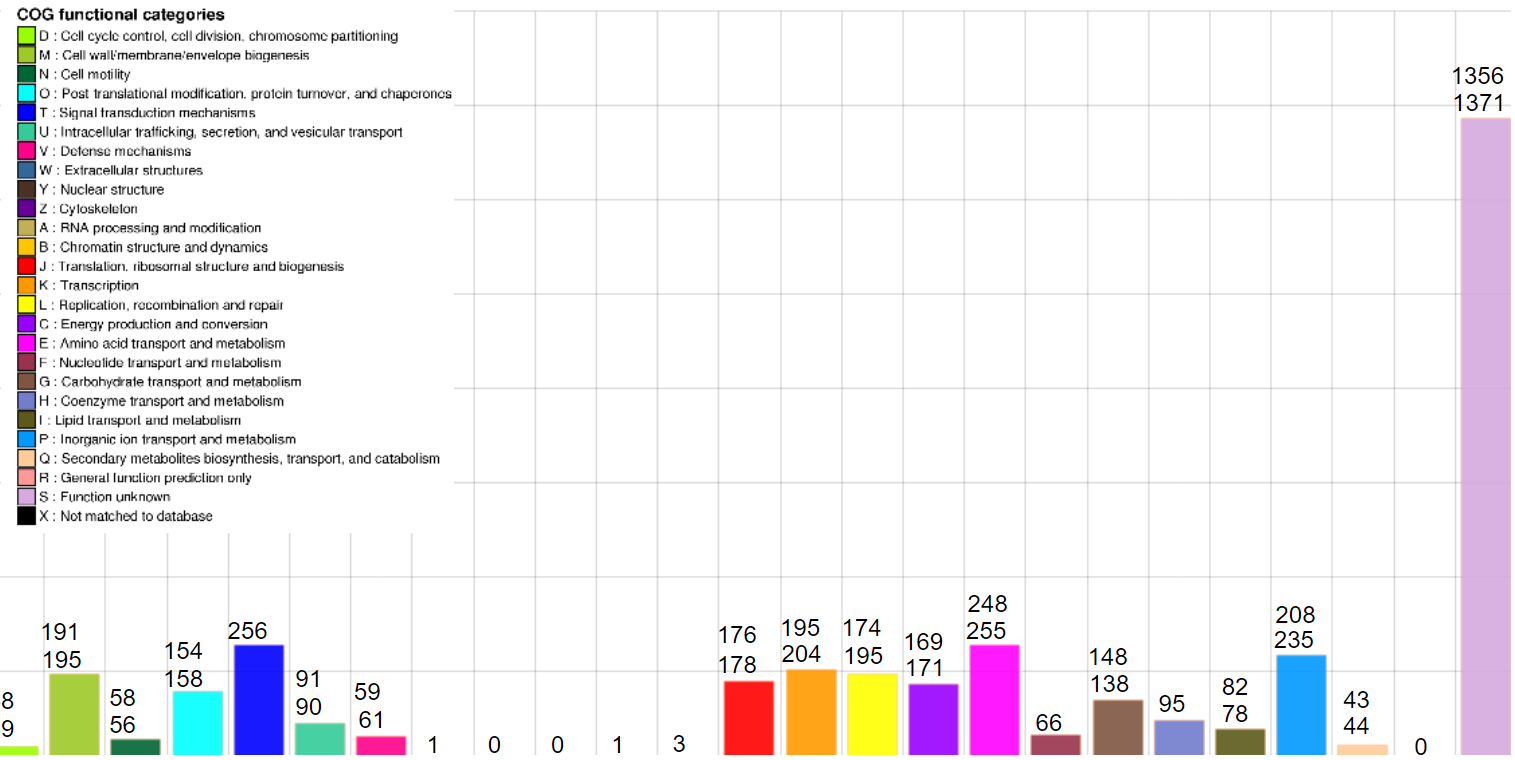


**Supplementary Figure 4.** Distribution of COG functional categories in *Pseudoalteromonas* sp. 16-SW-7 (numbers above) and *Pseudoalteromonas distincta* ATCC 700518^T^ (numbers below) calculated by EzBioCloud comparative genomics (CG).

## 2.2. Supplementary Tables

**Supplementary Table 1.** Genomic features and genomic similarity of strain 16-SW-7 and two reference strains.

| **Feature** | **16-SW-7** | ***Pseudoalteromonas distincta***  **ATCC 700518^T^** | ***Pseudoalteromonas paragorgicola***  **DSM 26439^T^** |
| --- | --- | --- | --- |
| **Genome features** |  |  |  |
| Genome size (bp) | 4,531,445 | 4,532,748 | 4,322,351 |
| No. of contigs | 2 | 32 | 22 |
| Size of circular chromosomes | 3,735,685  795,760 | - | 697863 |
| Size of the largest contig | 3,735,685 | 916,460 | 787,813 |
| G+C content (%) | 39.3 | 39.2 | 39.2 |
| Total genes | 4,025 | 4,077 | 4,206 |
| CDSs with protein | 3,858 | 3,871 | 3,930 |
| Genes assigned to COGs (Clusters of Orthologous Groups) | 3,192*  2,367** | 2,489** | - |
| Genes assigned to Metabolic Subsystems (RAST) | 1,359 | 1,400 | 1,282 |
| rRNAs | 28 | 29 | 11 |
| tRNAs | 103 | 99 | 109 |
| ncRNAs | 4 | 1 | 4 |
| Pseudogenes | 32 | 77 | 152 |
| **Genomic similarity** |  |  |  |
| ANI value (%) | 100% | 98.2 | 98.2 |
| dDDH value (%) | 100% | 84.4 | 83.5 |
| AAI value (%) | 100% | 98.6 | 98.8 |

*- Calculated by IMG/M server; **- Calculated by EzBioCloud server

**Supplementary Table 2.** The average nucleotide identity (ANI) calculation with the use of OrthoANIu algorithm (Yoon et al., 2017)

| OrthoANIu Results | *KMM 701/*  ATCC 700518^T^ | *KMM701/*  KMM 3548^T^ | ATCC 700518^T^/  KMM 3548^T^ |
| --- | --- | --- | --- |
| OrthoANIu value (%) | 98.22 | 98.19 | 98.04 |
| Average aligned length (bp) | 2,955,027 | 2,709,885 | 2,641,731 |
| Genome A coverage (%) | 65.22* | 59.81* | 58.46*** |
| Genome B coverage (%) | 65.40** | 62.84** | 61.26**** |

*- The coverage by the query genome KMM 701 (GenBank assembly accession: GCA_005877035.1); **- The coverage by the comparative genomes *P. distincta* ATCC 700518^T^ (GCA_000814675.1) and KMM 3548^T^ (GCA_014918315.1); ***- The coverage by the query genome *P. distincta* ATCC 700518^T^ (GCA_000814675.1); ****- The coverage by the comparative genome KMM 3548^T^ (GCA_014918315.1)

**Supplementary Table 3.** Calculated DNA-DNA hybridization between the strains *Pseudoalteromonas* sp. 16-SW-7, *Pseudoalteromonas distincta* KMM 638^T^ and *Pseudoalteromonas paragorgicola* KMM 3548T

**Supplementary Table 4**. The comparison of metabolic reconstruction of *Pseudoalteromonas* sp. 16-SW-7 with metabolic reconstruction of *Pseudoalteromonas distincta* KMM 638^T^ and *Pseudoalteromonas paragorgicola* KMM 3548T on the base of their coding sequences

**Supplementary Table 5.** The comparison of metabolic reconstruction of *Pseudoalteromonas distincta* KMM 638^T^ with metabolic reconstruction of *Pseudoalteromonas* sp. 16-SW-7 and *Pseudoalteromonas paragorgicola* KMM 3548^T^ on the base of their coding sequences.

**Supplementary Table 6.** The comparison of metabolic reconstruction of *Pseudoalteromonas paragorgicola* KMM 3548^T^ with metabolic reconstruction of *Pseudoalteromonas* sp. 16-SW-7 and *Pseudoalteromonas distincta* KMM 638^T^ on the base of their coding sequences.
